# An Internet-Based Consumer Resource for People with Low Back Pain (MyBackPain): Development and Evaluation

**DOI:** 10.2196/16101

**Published:** 2020-03-31

**Authors:** Paul William Hodges, Jenny Setchell, Mandy Nielsen

**Affiliations:** 1 The University of Queensland Brisbane Australia

**Keywords:** back pain, patient education, patient internet portals, evidence-based health care, patient involvement, service user involvement

## Abstract

People increasingly use the internet to obtain information about health complaints, including low back pain (LBP). LBP is the leading cause of disability internationally, and outcomes are worsening. There is an urgent need for resources that aid improvement of outcomes. There have been calls to engage consumers in the development of resources, but this has rarely been implemented. MyBackPain is a website that was developed with extensive involvement of consumers to ensure that the resource meets their needs for content and presentation. This paper aimed to describe the multistep process undertaken to develop the MyBackPain website and provide an extensive evaluation of its impact. Development of MyBackPain involved 10 steps, many of which have been published in the academic literature. These steps included consultation regarding consumer needs, evaluation of existing internet resources, identification of key messages to be reinforced, identification of frequently asked questions, consensus for content, content development (including development of algorithms to guide tailoring of the user experience), development of consumer-focused evidence-based treatment summaries, development of descriptions of health care providers, and testing. Evaluation included qualitative examination of people’s interactions with the website and its effects on their daily lives and an ongoing randomized controlled trial of impact of use of the site on people’s LBP-related health literacy, clinical outcomes, and treatment choices. It is hoped that the website can aid in the reduction of the massive burden of LBP and provide a template for the development of resources for other conditions.

## Introduction

### Background

The most recent global burden of disease study confirmed that low back pain (LBP) is the leading cause of disability worldwide [1], with enormous individual and economic burden. LBP accounts for 30% of all chronic pain [2] and affects up to 80% of individuals at least once in their life [3]. Unnecessary and ineffective assessments and/or treatments and poor quality management contribute to much of this burden [4]. Although negative messages and beliefs can lead to worse outcomes [5], beliefs about LBP can be positively affected by evidence-based information [6,7]. Furthermore, empowering patients to make informed choices can assist them to engage successfully with health advice and reduce care needs [8]. Early education and access to the most effective treatments could reduce the excessive burden of LBP. This understanding provides a foundation for a resource for people with LBP to provide accurate information about their condition, to empower them to actively participate in managing it, to navigate treatments, and to understand the roles of different health care providers.

Despite clear clinical guidelines, research of primary care physicians indicates that most individuals with LBP do not receive evidence-based care [4] and best possible outcomes are not being achieved [4]. For instance, contrary to guideline recommendations, more than 25% of patients are referred for imaging [4] and less than approximately 20% of patients with new LBP receive advice and education, although this is universally recommended in guidelines [4]. Of particular concern is the fact that 20% of patients with LBP are inappropriately prescribed opioids [4]. This research strongly suggests a gap in translating identified best practice and disseminating evidence-based LBP information.

People increasingly use the internet to obtain information related to health conditions [9,10]. The capacity of the internet to provide tailored information in varied formats at a time and place of the user’s choosing makes it an ideal platform to educate and engage people with LBP in the management of their condition. Notably, people with LBP consistently express a desire for trustworthy information about their condition [11-14]. Internet-based resources could enable patients to become better informed about their condition and treatment options [15-18] to improve outcomes and guide appropriate use of health resources [16,19,20]. Enhanced health literacy as a consequence of access to high-quality internet resources could also lead to efficient use of clinical consultation time [17,19], enhance relationships between patients and clinicians [16], and shared decision making [19].

Unfortunately, most websites about LBP provide inaccurate information [21] and are consistently rated as *poor* in overall quality when evaluated against criteria developed from relevant guidelines and research [22-24]. Furthermore, the criteria used to evaluate websites have largely been based on perspectives of researchers or clinicians [24-26], with a foundation in traditional literature [22,27] or clinical practice guidelines [22,23], and have not considered patient perspectives [28]. There is increasing emphasis placed on consideration of perspectives and preferences of people with a condition [29]. Furthermore, the relevance and accessibility of material are improved by involvement of consumers in the development of health information resources [30]. Despite repeated recommendations for consumer involvement in the preparation of educational resources [31], there has been limited attention to consumers’ views regarding content and presentation [15,26,32-34].

### Objectives

This paper describes the process undertaken to develop a consumer-focused internet-based resource for individuals with LBP and the evaluation of its impact. Here, we describe the multistep process undertaken to develop the resource, with specific emphasis on the engagement of consumers, clinicians, and experts at each step, and the plan and preliminary outcomes from analysis of impact.

## Methods and Results

### Overview of Website Development

**Figure 1:**
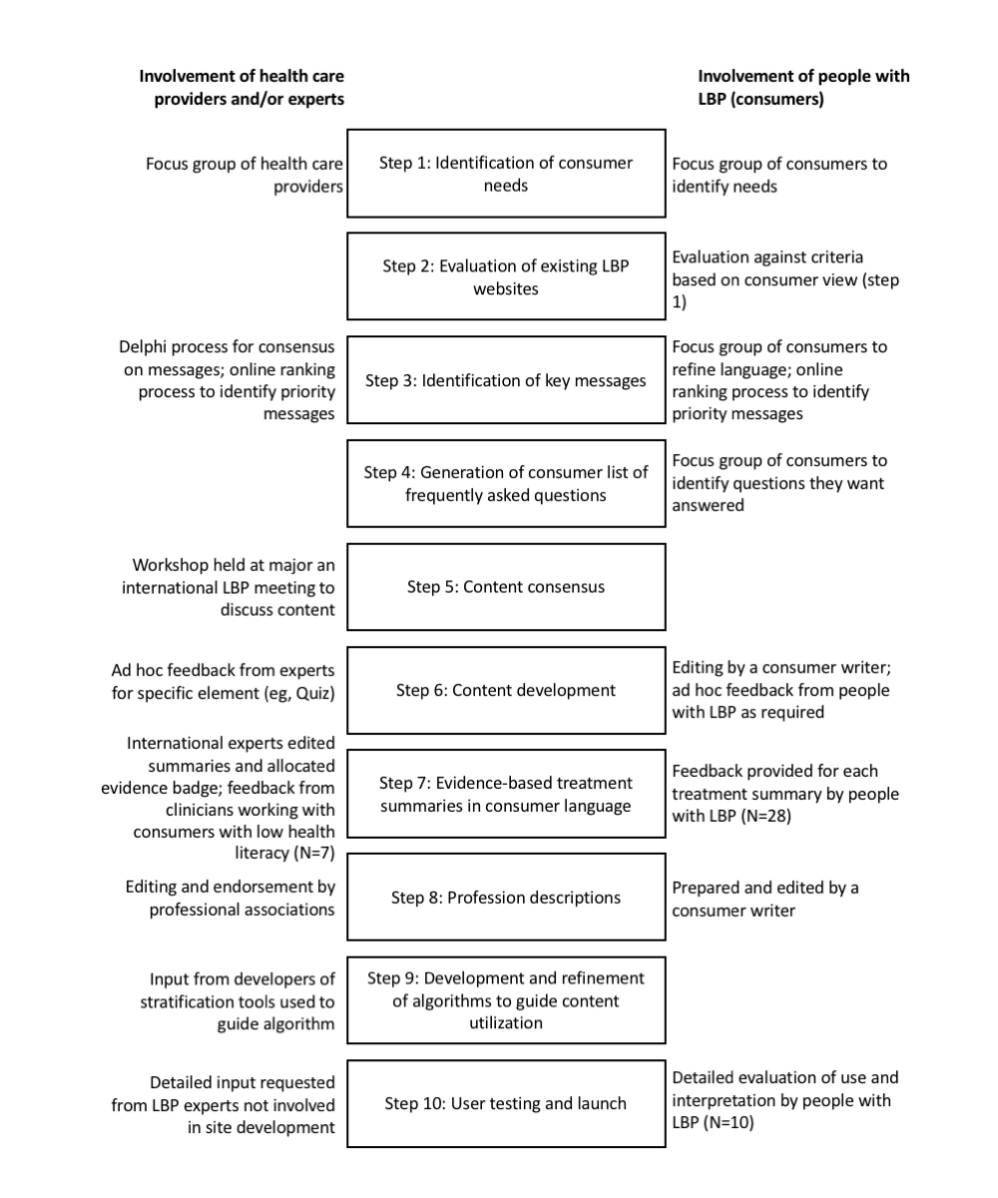
Steps undertaken for the development of the MyBackPain website. Involvement of consumers, health care providers, and experts is identified for relevant steps. LBP: low back pain.

The overall objective of the development of the internet resource for individuals with LBP was to provide high-quality, evidence-based resources that would meet the needs of consumers in terms of content and presentation. The overarching strategy was devised a priori and was planned to involve input from people with the condition (experts by experience), clinicians, experts from multiple disciplines, and professional societies at multiple time points (Figure 1). A series of research studies with qualitative and quantitative components were undertaken to inform the development and ensure the resource met the objectives.

### Development of an Internet Resource for People With Low Back Pain

#### Step 1. Identification of Consumer Needs—Website Content and Presentation

The first step in the development of the website was to undertake 2 qualitative studies to identify the needs of people with LBP in terms of content and presentation. These studies involved focus groups and interviews with people with LBP [35] and health care providers from multiple disciplines [36]. Data were analyzed thematically and used to generate a list of 12 content areas and 4 presentation preferences (an adapted version from Nielsen et al. [35] is presented in Textbox 1). Although people with LBP and health care providers agreed in most content areas, there were some differences. For instance, consumers wanted more specific explanation of diagnoses and treatments than the health care providers deemed possible or comfortable to provide on a website. Consumers also wanted capacity for consumer-to-consumer interaction in online forums, whereas health care providers express concerns that patient experiences may reinforce inappropriate messages. We deemed it too early to implement an online support group, and we have recently undertaken a systematic review (Maclachlan L, Mills K, Lawford BJ, Egerton T, Setchell J, Hall LM, Plinsinga ML, Besomi M, Teo PL, Eyles J, Mellor R, Hodges P, Hunter DJ, Vicenzino B, Bennell K, unpublished data, November 2019) and survey of views of individuals with musculoskeletal conditions [37] regarding online support groups as preliminary steps toward developing this component.

**Consumer preferences for website content and presentation.**

Desirable content:

1. Physiology/neurophysiology of low back pain—anatomy and explanation of pain
2. Different diagnoses
3. Treatment and management options (including alternatives)
4. Treatment outcomes (including side effects)
5. Roles of different medical and allied health professionals
6. Ways to improve function in daily life, eg, perform household tasks
7. The psychological and social impact of chronic low back pain
8. Ways to verify the quality of the information
9. Locally available health care resources/community groups
10. Product information
11. Questions to ask your health care practitioner
12. Information for partners and family members

Desirable presentation methods:

1. Mixture of presentation methods, including written information, videos, graphics, and animation
2. Interactivity
3. Lay person stories
4. Different levels of information—“drill down” if interested

#### Step 2. Evaluation of Existing Low Back Pain Websites

To determine the need for the potential resource, a review was undertaken to determine whether current websites already met consumer needs for content and presentation [38]. This review was undertaken at 3 different time points (2010, 2015, and 2019; to determine whether resources were changing over time) and involved virtual searches using Internet Protocol addresses from different countries (Australia, the United Kingdom, and the United States; to determine whether alignment of website content and presentation was better in some locations than others). All websites were evaluated against a 16-item checklist (12 content items and 4 presentation items) developed from the consumer preferences identified in step 1 [38]. The review identified that existing websites were poorly aligned with patient preferences, that this was not improving (or was even getting worse) over time, and did not differ between locations of the internet search. In 2010, no website was scored as *excellent*, and 58% scored as *poor* or *fair*. Key areas in which websites did not meet consumer needs were as follows: less than 50% of websites included information on treatment outcomes or information on psychological and social impact of chronic pain, less than 20% of websites included information regarding health care provider roles or questions to ask health care practitioners, and none of the websites included information for family and friends. On the basis of this review, it was deemed necessary to begin building a consumer-focused internet resource for people with LBP.

#### Step 3. Identification of Key Messages

In step 1, consumers and health care providers had expressed a clear preference for access to evidence-based information about multiple aspects of LBP. Although evidence for treatments and diagnostic procedures are frequently the subject of systematic reviews and meta-analysis, other aspects of advice and education are infrequently considered in this robust manner. As a result, we sought to identify and reach expert consensus on a list of evidence-based messages that should be reinforced frequently and consistently in various formats (eg, patient narratives and information sheets) in a website [39]. For this step, evidence-based messages were first identified from the literature (clinical practice guidelines and systematic reviews, eg, the study by Koes et al [40]), which produced a provisional list of 44 messages. Second, a multidisciplinary panel of experts and patients with LBP were consulted using a Delphi process to review, to add to, and to refine the key messages. Third, using consumer focus groups and a consumer writer, messages were refined and language was optimized to ensure messages were understandable and nonambiguous to people with LBP. This process resulted in a final list of 30 key messages that were categorized into 6 major thematic areas: principles of management, reassurance, staying active, unnecessary interventions, red flags, and disease knowledge (Table 1).

**Table 1:** Key messages identified for reinforcement throughout the website.

Thematic area and key message		Expert rank	Patient rank
**Stay active**			
	Bed rest for more than a day or two is not good	14	22
	Do not take back pain lying down	19	25
	Staying active helps prevent long-term back problems	5	8
	When you have back pain, carry on with normal activities as far as possible	2	24
	When you have back pain, staying active is important. You need to pace yourself to return to your usual activities	1	5
**Unnecessary investigations**			
	Blood tests are usually not needed in the majority of cases of LBP^a^	29	28
	CT^b^ scans have little use in diagnosing back problems, and caution should be exercised because of the large amount of radiation involved with their use	22	30
	Imaging (eg, x-ray, CT scan, or magnetic resonance imaging) is usually not needed in the majority of cases of LBP, particularly when your pain has been present for less than 6 weeks. Talk to your doctor about this	10	21
	X-rays will not highlight the cause of pain in most cases, unless a fracture is suspected	12	27
**Principles Mx^c^**			
	Health practitioners can assist in screening for causes of back pain	30	15
	If you have any further questions to ask your health practitioner, write them down and discuss them at your next visit	28	9
	Persistent LBP is influenced by a number of factors—physical, emotional, and environmental; it is important to address each of these areas	18	6
	Staying positive is important. Help is available	21	13
	Take ownership of your own well-being	20	20
	Work toward returning to your usual activities, with guidance from your health practitioner	16	7
	Work with your health practitioner to address your concerns	26	10
	Work with your health practitioners to manage your back pain	27	11
	Work with your health care team to set goals	25	16
**Disease knowledge**			
	In around 95% of cases, it is not possible to pinpoint the cause of back pain	23	29
	LBP may happen again over time	24	19
**Reassurance**			
	In most cases of recent onset back pain, the pain will get better in several weeks; however, this varies from person to person	7	14
	It is normal to worry about the cause of your back pain and the impact it may have on you	17	12
	It is not necessary to know the specific cause of your back pain to manage the pain effectively	13	23
	It is rare for LBP to be caused by a more serious health problem	9	26
	Most people find that their back pain settles down over a short period of time. If your back pain persists and is worrying you, consult a health professional	6	4
	Most people have pain in their low back at some stage in their lives	11	18
	Your pain may not necessarily be related to the extent of damage in your back. Hurt does not necessarily mean harm	3	17
**Red flag**			
	You should see a health practitioner if you have back pain and any of the following: pain that spreads down 1 or both legs: a fever, recent invasive procedure (eg, surgery), recent significant trauma, unexplained weight loss, and history of cancer	8	3
	You should see a health practitioner urgently if you have back pain and either of the following: bladder and/or bowel disturbance or significant leg muscle weakness	4	1
	You should see your health practitioner if your back pain is severe and it is worrying you, if you are having difficulty managing your back pain, or if your pain is getting worse	15	2

^a^LBP: low back pain.

^b^CT: computerized tomography.

^c^Mx: management.

Once developed, the list of key messages was subjected to 2 additional analyses. First, 2 groups (people with LBP and multidisciplinary international LBP experts) were asked to rank the messages in terms of their perceived priority or importance using an online process. This process highlighted some similarities in order of importance expressed by these 2 groups (eg, both groups prioritized messages related to identification of *red flags* to recommend the seeking of advice from a health care provider) but also some major differences. For instance, people with LBP prioritized messages about management strategies and ranked advice to avoid unnecessary investigations very low, whereas health care providers prioritized advice to stay active and reassurance. Contrasting views of experts and consumers were not unexpected but highlighted that care would be required to ensure patients were engaged with the website (eg, access to the information they wanted), and they were also guided to advice that may be contrary to their desires/beliefs (eg, patients continue to demand investigations despite evidence that they are only indicated in a small proportion of cases and evidence that early imaging can increase disability and duration of LBP [41]).

Second, developers of clinical practice guidelines that were published after the preparation of the list of key messages were surveyed using a Delphi method to ascertain whether messages remained consistent with newly developed guidelines. All messages were endorsed using this process.

#### Step 4. Generation of Consumer List of Frequently Asked Questions

In parallel with the development of a list of evidence-based key messages, a qualitative study with focus groups of people with LBP was undertaken to identify a provisional list of questions that consumers would like to have answered with an education resource (Nielsen M, Hodges PW, unpublished data January 2017). Thematic analysis of the focus groups provided an initial list of “frequently asked questions” (FAQs) to serve as a starting point for FAQs to be added to the site during development.

#### Step 5. Content Consensus

After establishing the needs of consumers and confirmation that a new resource was needed to meet these needs (described above), we held a workshop at the 13th International Back and Neck Pain Research Forum in Campos do Jordao, Brazil, to generate expert consensus on the plans for content of the website. At the workshop, participants endorsed the findings of the prior steps, and it was agreed that the website should aim to improve health outcomes for individuals with LBP by (1) enhancing the confidence of individuals with LBP to manage their condition and make evidence-based treatment choices and to avoid ineffective, unnecessary, and potentially harmful investigations and treatments; (2) demedicalizing and normalizing LBP with messages in multiple formats that reinforce that LBP is a natural part of life for many and in most cases can be managed with early return to activity; (3) providing tools for individuals to identify whether further investigation and/or management may be required; and (4) engaging patients in healthy behaviors and attitudes about LBP. These objectives of the website were used to underpin the development of the content in step 6.

#### Step 6. Content Development

Using the outcomes from steps 1 to 5 as a starting point, an extensive process of content development was undertaken with leadership by a postdoctoral research fellow (background in physiotherapy and psychology), an international multidisciplinary steering committee, and a consumer writer. A partnership was developed with Arthritis Australia, which is a charitable, not-for-profit organization and the peak arthritis consumer body in Australia, to host the site and aid in the development of specific areas of content (eg, preparation of videos).

Table 2 presents the final list of content areas and the intended purpose of each. Content was developed using varying methods and consultations according to nine key principles: (1) written in consumer language, (2) evidence based, (3) bio-psycho-social underpinning, (4) aligned with key messages, (5) no commercial goal, (6) no single professional affiliation/bias, (7) focus on empowerment of patient to take control of their LBP, (8) focus on reassurance and informed choices, and (9) using a design that would be engaging for users (contemporary, interactive, and intuitive). Content presentation methods were aligned with the preferences of consumers identified in step 1. Development of content for several areas required a more detailed process and are described separately in steps 7 to 9.

**Table 2:** Final content developed for MyBackPain website.

Content area		Purpose	
Guide me		Provide tailored guidance regarding prognosis and recommended resources based on responses to questions based on 2 evidence-based prognostic tools	
**Back pain information**			
	About back pain		General information regarding back pain with emphasis on reassurance and bio-psycho-social model of pain
	Do it yourself		Summary of useful tips to help people with LBP^a^ to do the things they want/need to do. Also link to other online self-help tools (eg, *Paintrainer* program to learn pain coping skills)
	Treatments		Evidence-based summaries of 80 common treatments for LBP in consumer-friendly language. Evidence badges to provide quick reference of efficacy of intervention. Information of how to prepare for a consultation with a health care professional and questions to ask
	Health care professionals		Description of health care professionals who commonly manage LBP and how to find them
	Test your knowledge		Quiz to test knowledge about LBP that addresses main key messages and common myths about LBP
	For family and friends		Guide to information for family and friends to understand LBP and provide support
**Videos**			
	Back pain information		Library of videos designed to provide narratives that reinforce key messages
	Living well with back pain—people’s stories		Stories of people who are living with LBP
**Frequently asked questions**		Key questions identified by consumers as issues they want to understand better	
	What can I do to help my LBP?		Response to questions related to self-management
	Back pain causes		Responses to questions related to back pain causes
	What is going to happen?		Responses to questions regarding prognosis and other requests
	Seeking help		Responses to general questions regarding health professionals
About us		Information regarding the developers and funders of the MyBackPain website	
**Other features**			
	Daily/weekly healthy messages by email		Messages sent to users, based on key messages, sent at a frequency indicated by the user

^a^LBP: low back pain.

#### Step 7. Evidence-Based Treatment Summaries in Consumer Language

**Figure 2:**
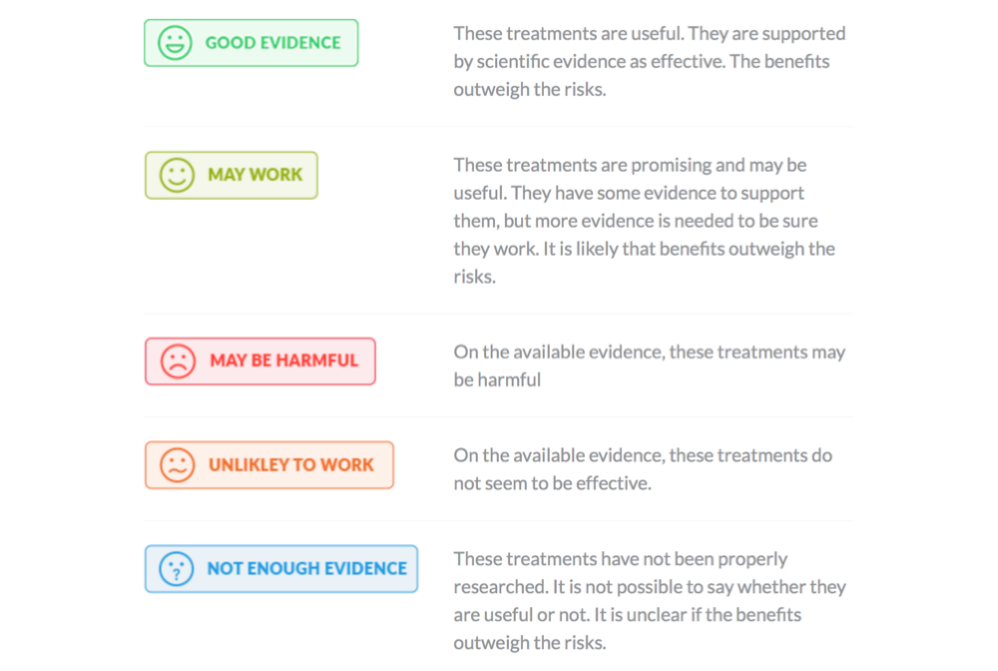
Evidence grading badges developed to enable identification of evidence levels for treatments or the potential for harm.

Orthodox and complimentary treatments commonly used by people with LBP were identified by the expert steering committee with consumer input. The committee agreed upon a final list of 80 treatments grouped into 16 broad areas (Table 2). An independent expert group (International Centre for Allied Health Evidence, University of South Australia, Adelaide, Australia) was contracted to develop a draft description of each treatment, and a synthesis of research evidence was prepared from the best available evidence (systematic reviews, clinical trials, and clinical practice guidelines). Draft descriptions were edited for language by a consumer writer. For each treatment, information was provided regarding (1) basic description (what is it?, how does it work?, and is it effective for treating back pain?), (2) detailed treatment information, (3) points to consider (defined as “pluses” and “minuses”), (4) FAQs, and (5) key references. A series of “evidence grade badges” was developed by the expert steering committee with consultation with external experts in evidence-based practice and a consumer writer (Figure 2). Evidence grades aimed to enable quick identification of evidence levels for treatments or the potential for harm and were also designed not to overemphasize scientific evidence (or lack of) as the only source of information that might be of value to consumers. International experts in each type of treatment (including a senior and early career researcher where possible) were identified by the expert steering committee to review each treatment summary and allocate an “evidence grade badge.” All summaries and evidence grades were reviewed for consistency by the steering committee and 3 additional experts over a series of teleconferences. After completion of the 80 treatment summaries, 28 individuals with LBP were recruited to provide detailed review of 2 to 3 treatment summaries each to provide feedback of content and language. We also garnered feedback from 7 clinicians working with people likely to have low health literacy.

#### Step 8. Profession Descriptions

In step 1, consumers had indicated confusion regarding the role of different health care providers. Descriptions were prepared for health care providers who manage back pain that have a registration board in Australia. Descriptions were prepared by the expert steering committee and consumer writer. These were refined and then approved by the professional associations that represent each discipline.

#### Step 9. Development/Refinement of Algorithms to Guide Content Utilization

A total of 2 algorithms were developed on the basis of existing stratification/prognostic tools to guide users of the website toward information that is tailored to their individual needs. The STarT Back tool [42] and Pick-up tool [43] were adapted to guide the user experience for individuals with LBP of greater than or less than 3 months duration, respectively. The STarT Back tool stratifies individuals with low, medium, and high risk for poor prognosis based on responses to 9 questions. The Pick-up tool calculates probability of good outcome based on responses to 5 questions. The tools were used to evaluate possible risk of poorer outcome and tailoring information regarding advice, particularly with respect to providing reassurance, and recommendations for access to psychologically informed resources were necessary. The “guide me” algorithm also included identification of *red flags* (eg, change in bladder and bowel function and perineal numbness) to trigger advice to seek medical consultation.

#### Step 10. User Testing and Launch

**Figure 3:**
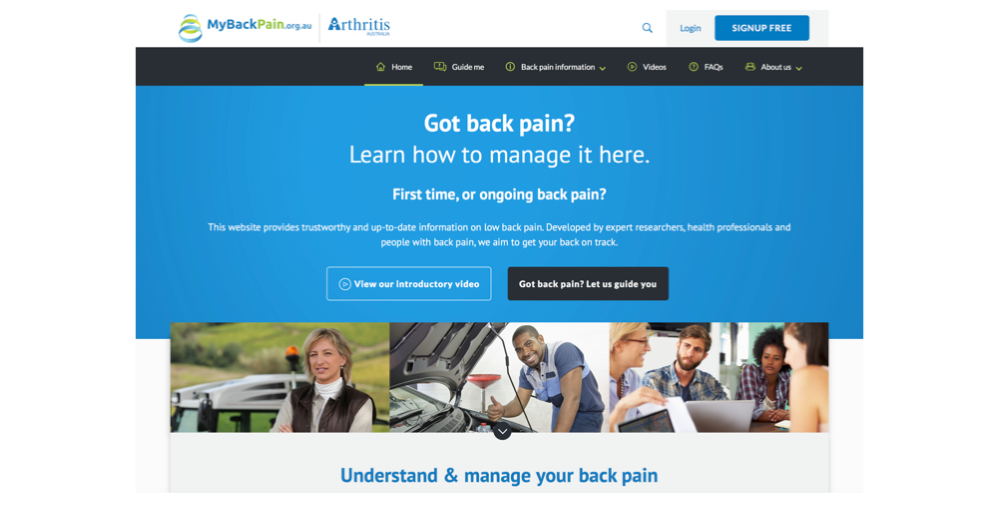
Screenshot of landing page for MyBackPain, an internet resource designed for people with low back pain to obtain information about their condition and guidance for managing/living with low back pain.

A full beta version of the website was constructed and extensively reviewed by consumers and experts. In-depth consumer input user testing was conducted with 10 individuals with LBP of different presentations and durations. Each consumer was observed as they interacted with the website and asked to voice what they were thinking as they moved through the site. This testing focused on both site content and functionality. A summary of feedback was recorded. Four experts who were not involved in development of the site were asked to provide detailed review of the website and written feedback. All feedback from the consumers and experts was discussed by the expert steering committee and postdoctoral fellow and addressed if appropriate. The site was launched on the July 30, 2019 [44]. The launch date was 12 months after the website was completed to ensure that the primary end point for a randomized controlled trial (RCT) of the impact of use of the website (see below) was not affected by control group participants inadvertently accessing the site. Figure 3 shows the landing page of the website. Multimedia Appendix 1 is a brief video that was prepared for people with LBP and clinicians who treat LBP to outline the purpose and content of the website.

### Evaluation of MyBackPain

#### Evaluation 1: Randomized Controlled Trial of Impact of MyBackPain

Before the launch of MyBackPain, an RCT was undertaken to investigate the effectiveness of the website in improving spinal health literacy, treatment preferences, and clinical outcomes for people with LBP, in comparison with other online resources. MyBackPain was made available by username/password access only during this period. The pragmatic trial was conducted online. Participants were 440 people with nonspecific LBP of any duration, stratified to those with LBP for a duration of greater than or less than 12 weeks. Participants, research staff, and the biostatistician were blinded to treatment allocation. Data were collected at baseline and 1, 3 (primary end point), 6, and 12 months via online surveys and questionnaires. The primary outcome measure was spinal health literacy measured using dimensions 2 and 3 (“having sufficient information to manage my health” and “actively managing my health”) of the Health Literacy Questionnaire [45]. Participants are asked to consider their LBP when answering the survey. Secondary outcomes include the quality of treatment preferences (whether patients choose treatments that are supported by evidence) and LBP clinical outcomes (pain, disability, and quality of life). The trial has been prospectively registered (ACTRN12617001292369; registered on September 7, 2017). Long-term outcomes will be finalized in May 2020, with trial results available soon thereafter.

#### Evaluation 2: Interdisciplinary, Postqualitative Evaluation of MyBackPain

Although health websites provide information in a convenient format, they can be reductionist in their capacity to accommodate the complexities of human life, health, evidence, and the diverging philosophies underpinning different forms of health care. A postqualitative analysis of MyBackPain was undertaken by a team with backgrounds in public health, sociology, physiotherapy, psychology, and occupational therapy and an *expert consumer* with LBP (Setchell J, Olson R, Turpin M, Costa N, Barlott T, O’Halloran K, Wigginton B, Hodges P, unpublished data, February 2019). The analysis aimed to evaluate the success of the website at providing health information that was simultaneously *scientifically rigorous* and avoidant of associated pitfalls such as reduced consideration of complexity of the condition. The analysis was guided by Ahmed’s theory [46] of the socioculturality of emotions and was designed to reflect on experiences as the team individually navigated the website, followed by team discussion. Through this postqualitative inquiry process, it was recognized that some forms of communication used in the website had the potential to marginalize some users (eg, although images showed individuals of diverse background, all were happy and undertaking productive activities, which may marginalize users who do not identify with those images, and *evidence* for treatment was limited to RCTs—although this is conventionally used in most evaluations of treatment efficacy, other forms of evidence [eg, qualitative interpretations] can contribute and can be desired by users) but liberated possibilities of others (eg, videos presented real stories by real people). Caution was identified regarding the assumption that consumer education and *choice* enhance consumer health and the potential unintended negative effects of the focus on changing individual behaviors, particularly *lifestyle* factors such as activity and exercise, which can lead to feelings of guilt when this cannot be achieved and shame that they are somehow responsible for their LBP. Each of these issues can be addressed though refinement of website messaging and explicit recognition within the website to acknowledge the issues.

#### Evaluation 3: Qualitative Analysis of People’s Interactions With the Website and Its Effects in Their Daily Lives

A qualitative study was undertaken using methods adapted from discourse analysis to identify potential *tensions* in the website content (Setchell J, Turpin M, Costa N, Hodges P, unpublished data October 2019). Participants with LBP were observed while interacting with the website and asked to discuss their responses. For 1 month before a follow-up interview, these participants took photographs of what was happening in their lives when they thought of the website. Photographs were used to prompt discussion. A postcritical discourse analysis approach identified 4 areas of *tension* in the presentation of material on the website: (1) the website focused on reducing LBP, with little discussion of living with LBP, which may be the goal or the reality for some individuals; (2) the website tended toward discussion of keeping active and not resting, potentially leading to feelings of guilt if activity targets could not be met; (3) there was tension between educating people with LBP to make their own choice vs providing explicit guidance, with the desired balance between these depending on the individual user; and (4) although the treatment summaries intend to inform users of evidence-based treatments to guide choices, this information had an unintended negative impact on some participants who had used disproven or potentially harmful treatments. These tensions were unanticipated in the design of the website and will be addressed by explicit recognition and discussion of these *tensions* in multiple formats (eg, videos and pop-up boxes at appropriate locations of the site where tensions arise).

#### Plan for Review and Revision of MyBackPain

As information regarding LBP (particularly treatments) evolves with future research, there will be a need to review and update the content of the website. A governance structure has been established to overview regular review and revision of the content. The website uses a content management system with concurrent possibilities for updating much of the content. Critically, the content of the treatment summaries will be updated at least biannually with the guidance of the international advisors who contributed to their development (and others, as appropriate). New content is planned and contingent upon future funding.

## Discussion

This paper describes the multistep process undertaken to develop a website for people with LBP to meet their expressed needs for content and presentation. The rigorous process used to develop this resource is rare, and we hope that outlining the iterative steps we undertook might help others to develop resources for consumers. A major component of the process was extensive involvement of consumers in defining the content, providing feedback on the content, and evaluating the final website. A multidisciplinary group of international experts were recruited to guide development of the website and provide input/review of content at many steps. Most steps involved in the development of the website were undertaken in a formal manner with publication and presentation of the results in academic literature.

MyBackPain was designed to address the issue that most available resources do not meet the expressed needs of people with LBP [38] and use language that is not optimized for users to understand [23]. It has also been identified that websites for LBP generally provide inaccurate information and do not consider the spectrum of presentations of LBP [21], another key objective of the development of MyBackPain.

It is hoped that MyBackPain will provide a useful resource for people with LBP and their friends and families. We also hope that health care providers will derive benefit from referring patients to the website for reinforcement of key messages and to generate a partnership in decision making for treatment. Ultimately, the intention of the website is to contribute to reducing the massive burden of LBP. The extensive process of development and consumer/expert engagement that we have undertaken could also provide a template for the development of resources for other conditions.

## Appendix

Multimedia Appendix 1 Explanatory video for MyBackPain: introduction to purpose and features of the website.

## Abbreviations

FAQs: frequently asked questions
LBP: low back pain
RCT: randomized controlled trial
